# LONG-TERM COSTS OF GRANDPARENT CAREGIVING: RESULTS FROM THREE WAVES OF THE MIDLIFE IN THE UNITED STATES STUDY (MIDUS)

**DOI:** 10.1093/geroni/igz038.2936

**Published:** 2019-11-08

**Authors:** Elizabeth Rickenbach, Elizabeth H Rickenbach, Chih-Chien Huang, Jessica Y Allen, Kelly E Cichy

**Affiliations:** 1 Saint Anselm College, Manchester, New Hampshire, United States; 2 Birmingham-Southern College, Birmingham, Alabama, United States; 3 Kent State University, Kent, Ohio, United States

## Abstract

Cross-sectional studies reveal the health burden of grandparent caregiving. Still, longitudinal, research is needed to understand how grandparent caregiving compromises grandparents’ long-term health. Using three waves of data from the Midlife in the United States Study (MIDUS), we examined sociodemographic factors, health and well-being outcomes between caregiving (CG) and non-caregiving (NCG) grandparents. By wave 3, 12.8% (n = 234) were CG. CG were younger, more likely female, and had lower income and education. MANCOVA adjusted for age, gender, education, and number of children revealed CG reported poorer physical and emotional well-being (e.g. higher depression, anxiety, lower life satisfaction, greater morbidity); CG were consistently less healthy than NCG across all three waves. Lower income and less healthy older adults are more likely to become grandparents, and they remain less healthy over time. Policies and resources to assist grandparents, particularly low-income and vulnerable older adults who are caring for grandchildren, are needed.

